# Myeloperoxidase and N-terminal proatrial natriuretic peptide as predictors for atrial fibrillation recurrence in patients undergoing redo ablation

**DOI:** 10.1016/j.hroo.2024.09.003

**Published:** 2024-09-13

**Authors:** Marwin Bannehr, Christian Georgi, Christoph Edlinger, Vera Paar, Paulina Jankowska, Michael Lichtenauer, Anja Haase-Fielitz, Martin Seifert, Christian Butter

**Affiliations:** 1 Department of Cardiology, University Hospital Heart Centre Brandenburg, Brandenburg Medical School (MHB) Theodor Fontane, Bernau/Neuruppin, Germany; 2 Faculty of Health Sciences, Joint Faculty of the University of Potsdam, the Brandenburg Medical School Theodor Fontane, and the Brandenburg Technical University Cottbus-Senftenberg, Cottbus, Germany; 3 Department of Cardiology, Clinic of Internal Medicine II, Paracelsus Medical University of Salzburg, Salzburg, Austria; 4 Institute of Social Medicine and Health System Research, Otto von Guericke University Magdeburg, Magdeburg, Germany

**Keywords:** Atrial fibrillation, Ablation, Fibrosis, MPO, NT-proANP, Biomarkers, Low-voltage areas

## Abstract

**Background:**

Atrial fibrillation (AF) is a progressively developing arrhythmia. Electroanatomic remodeling may play an important role, both in the development of the disease and in the perpetuation and thus in the recurrence of AF.

**Objective:**

This study aimed to investigate potential biomarkers myeloperoxidase (MPO), N-terminal proatrial natriuretic peptide (NT-proANP), intercellular adhesion molecule-1, and matrix metalloproteinase-2 and their predictive value for AF recurrence in patients undergoing redo ablation.

**Methods:**

In this single-center prospective cohort study, 50 consecutive patients underwent ultra high-density mapping and redo ablation. Biomarkers were determined before ablation and at 6-month follow-up. Seven-day Holter was conducted to check for AF recurrence (>30 seconds).

**Results:**

Eleven (22%) patients showed AF recurrence after redo ablation. Receiver-operating characteristic curve analysis revealed venous MPO and NT-proANP (area under the curve [AUC] 0.755, 95% CI 0.599–0.912, *P =* .010; and AUC 0.752, 95% CI 0.551–0.953, *P =* .011) as acceptable predictors for intermediate AF recurrence, whereas matrix metalloproteinase-2, intercellular adhesion molecule-1, and established cardiovascular biomarkers such as N-terminal pro–B-type natriuretic peptide, troponin T, and C-reactive protein were not (all AUC <0.600). MPO and NT-proANP correlated with AF burden (ρ = 0.365, *P =* .005; and ρ = 0.362, *P =* .005). While MPO was associated with atrial fibrosis in the endocardial map (ρ = 0.280, *P =* .024), NT-proANP correlated with left atrial volume index (ρ = 0.256, *P =* .037). There were no significant differences in biomarkers concentrations with regard to venous and coronary sinus samples.

**Conclusion:**

MPO and NT-proANP are of predictive value for AF recurrence in patients undergoing redo ablation. While MPO correlated with endocardial fibrosis, NT-proANP was associated with left atrial volume.

## Graphical abstract

**Figure:**
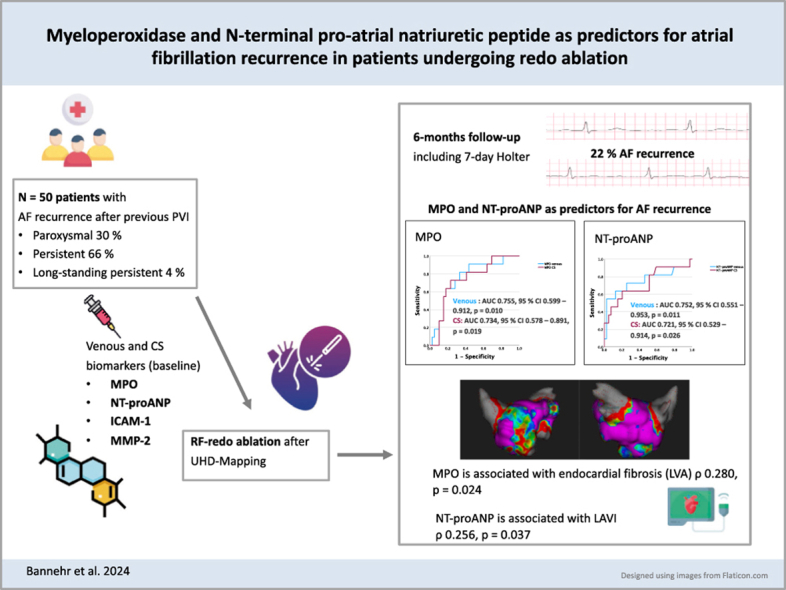

**Key findings**

▪ Myeloperoxidase (MPO) and N-terminal proatrial natriuretic peptide (NT-proANP) are of predictive value for atrial fibrillation recurrence in redo ablation.
▪ MPO correlates with left atrial endocardial fibrosis.
▪ NT-proANP is associated with left atrial volume.
▪ No differences were found in MPO and NT-proANP levels when comparing venous and coronary sinus samples.

## Introduction

Atrial fibrillation (AF) is a progressively developing arrhythmia. It may be triggered by spontaneous premature depolarizations of myocytes originating from the pulmonary veins, which can be treated by pulmonary vein isolation (PVI).^1^ However, a significant number of patients, particularly with persistent and long-standing persistent AF, remain refractory to this approach, pointing toward involvement of atrial tissue as a substrate in those types of AF.^2^

This may be the reason why success rates of catheter ablation in AF, with PVI as cornerstone, often are not satisfactory. Patients therefore often undergo multiple procedures.

Atrial electroanatomic remodeling has been shown to play an important role both in the development of the disease and in the perpetuation and thus in recurrence of AF. Fibrosis-driven AF implies disturbance in electrical conductance because of disruptions in intercellular communication of atrial myocytes. Collagen fibers represent electrical barriers, which can cause asynchronous propagation of electrical activation.^3^

The pathogenesis of atrial fibrosis is multifactorial and involves resident cardiac cells as well as infiltrating leukocytes, generating and sequestering matrix metalloproteinases (MMPs). A growing body of evidence has shown an important role of reactive oxygen species in the release and activation of pro-MMPs and the stimulation of profibrotic cascades. Myeloperoxidase (MPO), a bactericidal enzyme released from activated neutrophils, catalyzes the generation of reactive oxygen species, which affect intracellular signaling in various cells, including myocytes. Thus, MPO advances the activation of pro-MMPs as well as the deposition of atrial collagen, possibly resulting in atrial arrhythmias.^4^ Furthermore, endothelial adhesion molecules are expressed in activated endothelial cells, including the atria. Among those, intercellular adhesion molecule-1 (ICAM-1) expression is increased in inflamed endothelium and responsible for the adhesion and migration of monocytes and lymphocytes.^5^ The current literature suggests alterations in ICAM-1 tissue expression in patients with AF.^6^^,^^7^

Underlying diseases, such as hypertension or heart failure, increase atrial stress, leading to left atrial (LA) dilatation, a potential risk factor for atrial remodeling. N-terminal-proatrial natriuretic peptide (NT-proANP) is predominantly released in the atrial myocardial wall as a response to atrial wall stretch.^8^ Elevated natriuretic peptide levels were found to be associated with an increased risk of AF initiation.^9^ Local voltage abnormalities are considered a surrogate marker for fibrosis. They can be quantified and localized via high density endocardial electroanatomic mapping during an invasive electrophysiologic study.^10^ Low-voltage areas (LVAs) are considered both a predictor of arrhythmia recurrence and a frequent target in AF ablation, although the added value of the latter is uncertain.^11^, ^12^, ^13^ Other means of evaluating local areas of fibrosis are cardiac magnetic resonance imaging (late gadolinium enhancement), which is limited in its spatial resolution, time-consuming, and rather costly, or histopathological analysis, which naturally comes with a sampling error and is always invasive.^14^^,^^15^

Blood biomarkers are widely used in epidemiological and clinical studies to refine risk assessment of AF.^16^ Several biomarkers have been shown to be elevated in AF and may be involved in underlying pathophysiological processes such as inflammation and fibrosis. However, recent studies about LVA prediction and recurrence are inconclusive.

### Purpose

This study was performed to assess whether the preablation plasma levels of MPO, NT-proANP, ICAM-1, and MMP-2 are predictive of AF recurrence in patients undergoing redo ablation.

## Methods

### Patient population

In this single-center prospective cohort study, 50 consecutive patients with symptomatic recurrence of paroxysmal, persistent, or long-standing persistent AF who had already undergone PVI, cryoballoon, or radio frequency ablation, were enrolled. Patients were admitted at a tertiary care center and scheduled for redo ablation between January 2020 and January 2023. Patients with known autoimmune disease, pulmonary fibrosis, and chronic obstructive pulmonary disease GOLD (Global Initiative for Chronic Obstructive Lung Disease) ≥2 were excluded. Liver elastography was performed to rule out liver fibrosis.

Demographics, clinical characteristics, comorbidities, procedural characteristics, electrocardiogram at baseline and follow-up, and echocardiographic findings were obtained from the electronic medical recording system by expert staff blinded to the aim of the analysis. The study was approved by the local ethics committee of Brandenburg Medical School Theodor Fontane (E-01-20200106) and conducted according to the Declaration of Helsinki. Written informed consent was obtained from all participants.

### Endpoints

The primary endpoint was recurrence of AF defined as atrial tachycardia (AT)/AF >30 seconds in 7-day Holter or cardioversion within the study period after 3 months of blanking.

The secondary endpoints were AF burden and presence of LA fibrosis in the endocardial ultra high-density map, as well as an association with left atrial volume index (LAVI).

Differences in biomarkers before and after ablation were determined. The predictive value of biomarkers for the primary endpoint and correlation with the secondary endpoints were investigated.

### Ultra high-density mapping and ablation procedure

All patients underwent ultra high-density mapping using the Orion multielectrode basket mapping catheter (Boston Scientific) and the Rhythmia mapping system (Boston Scientific).

LVAs were defined as sites with a bipolar peak-to-peak voltage of <0.5 mV with an extent of >1 cm^2^, as described previously.^17^, ^18^, ^19^ The previous Association of atrial tissue fibrosis identified by delayed enhancement MRI and atrial fibrillation catheter ablation (DECAAF I) trial proposed a risk stratification (UTAH classification), with regard to AF recurrence after PVI, based on quantitative LA magnetic resonance imaging extent of late gadolinium enhancement.^14^ We applied the same cutoffs (<10%, ≥10% to <20%, ≥20% to <30%, and ≥30%) to our endocardial LVA data as surrogates for fibrosis. For this reason, LA surface area was measured, and low voltage was set in relation to the total surface area. Since all patients included in this study had undergone prior PVI, atria were cut manually at the anatomic pulmonary vein ostia and the mitral annulus with the help of the Rhythmia software’s cutting tool, and only the LA body was included in any surface area analysis.

The ablation strategy, in brief, consisted of checking pulmonary vein reconduction and closing detected gaps. Further ablation beyond reisolation of the pulmonary veins (cavotricuspid isthmus, LA lines, LVA ablation) was at the investigator’s discretion but in general dependent on the substrate in the electroanatomic voltage map and the critical isthmus of additional ATs.

### Biochemical assessment

Blood samples from a peripheral vein were collected during the index procedure (before ablation) and at 6-month follow-up. In addition, during the procedure a blood sample from the coronary sinus was taken via a 6F AL 2 catheter (Cordis). Coronary sinus sampling was performed to detect transcardiac gradients of circulating biomarkers that are primarily cardiac in origin. Immediately after collection, blood samples were transferred to EDTA tubes and centrifuged at 4 °C and 3,500 rpm for 15 minutes. The supernatant plasma was then aliquoted into three 800 μL portions per sample in 2 mL tubes and frozen at –80 °C until further processing. Biomarkers were analyzed using commercially available enzyme-linked immunosorbent assays (human MPO ELISA Kit DY3174, human NT-proANP ELISA Kit DY8247-05, human ICAM-1/CD54 ELISA Kit DY720, human MMP-2 ELISA Kit DY902 [all R&D Systems]).

### Follow-up

Seven-day Holter was conducted to check for AF/AT recurrence (episodes lasting longer than 30 seconds) at the 6-month follow-up visit. AF burden was defined as percentage in AF/AT during the respective time of Holter. Episodes were manually reviewed and confirmed, and only then added to AF burden. Electrocardiograms were analyzed by cardiac electrophysiologists.

### Statistical analysis

Statistical analyses were performed using SPSS 29.0 software (IBM). Continuous variables were reported as mean ± SD, if normally distributed, or median (interquartile range) if not normally distributed. Categorical variables were expressed as absolute numbers and/or percentages. Unpaired Student *t* test, Mann-Whitney *U* test, and the chi-square test were used when appropriate to test for differences between groups. Spearman correlation coefficients were calculated to measure the strength of association between variables. Receiver-operating characteristic curve analyses were performed to illustrate the diagnostic ability. Original data will be made available upon reasonable request.

## Results

### Clinical characteristics

Fifty patients were included in the study (58 enrolled, 1 dropped out, 7 were lost to follow up). Of those, 15 (30%) had paroxysmal, 33 (66%) persistent, and 2 (4%) long-standing persistent AF. Baseline characteristics are shown in Table 1.

**Table 1:** Baseline patient characteristics

	Overall (N = 50)	No recurrence (n = 39 [78%])	Recurrence (n = 11 [22%])	*P* value
Age, y	67.4 ± 8.5	67.9 ± 7.9	65.5 ± 10.2	.204
Male	30 (60)	15 (38.5)	5 (45.5)	.467
BMI, kg/m^2^	29.3 ± 5.4	29.5 ± 5.8	28.4 ± 3.5	.271
Type of AF				
Paroxysmal	15 (30)	9 (23.1)	6 (54.5)	.054
Persistent	33 (66)	29 (74.4)	4 (36.4)	.025
Long-standing persistent	2 (4)	1 (2.6)	1 (9.1)	.395
Number of previous ablations				.654
1	36 (72)	27 (69.2)	9 (81.8)	
2	12 (24)	12 (30.8)	0 (0)	
3	1 (2)	0 (0)	1 (9.1)	
4	1 (2)	0 (0)	1 (9.1)	
Coronary artery disease	13 (26)	9 (23.1)	4 ((36.4)	.301
Hypertension	45 (90)	35 (89.7)	10 (90.9)	.699
Type 2 diabetes	5 (10)	4 (10.3)	1 (9.1)	.699
Peripheral artery disease	2 (4)	0 (0)	2 (18.2)	.045
Previous stroke	5 (10)	4 (10.3)	1 (9.1)	.699
Obstructive sleep apnea	4 (8)	3 (7.7)	1 (9.1)	.643
Left ventricular ejection fraction, %	62.1 ± 6.3	61.8 ± 6.7	63.8 ± 3.1	.427
LAVI, mL/m^2^	83.8 ± 21.9	81.6 ± 23.7	91.5 ± 21.1	.190
LVA, cm^2^	46.7 ± 43.7	40.8 ± 39.5	71.9 ± 53.8	.053
Fibrosis in relation to total LA surface area				<.001
<10%	28 (56)	27 (69.2)	1 (9.1)	
≥10% to <20%	9 (18)	8 (20.5)	1 (9.1)	
≥20% to ≤30%	6 (12)	4 (10.3)	2 (18.2)	
≥30%	7 (14)	0 (0)	7 (63.6)	
PV reconnections	32 (64)	25 (64.1)	7 (63.6)	.977
CKD-EPI GFR, mL/min	78.1 ± 15.4	78.9 ± 14.9	75.0 ± 17.3	.461
Antiarrhythmic drugs∗	11 (22)	8 (20.7)	3 (27.3)	.529
Beta-blocker	48 (96)	37 (94.9)	11 (100)	.605
CHA_2_DS_2_-VASc score	3 (2–4)	3 (2–4)	3 (2–4)	.703

Values are mean ± SD, n (%), or median (interquartile range).

AF = atrial fibrillation; CHA_2_DS_2_-VASc = congestive heart failure, hypertension, age ≥75 years, diabetes mellitus, prior stroke or transient ischemic attack or thromboembolism, vascular disease, age 65–74 years, sex category; CKD-EPI = Chronic Kidney Disease Epidemiology Collaboration; GFR = glomerular filtration rate; LA = left atrial; LAVI = left atrial volume index; LVA = low-voltage area; PV = pulmonary vein.

∗ Amiodarone, flecainide.

### AF recurrence

Eleven (22%) patients developed AF recurrence during follow-up. Mean AF burden in those with recurrence in 7-day Holter was 55 ± 48%. Interestingly, we observed more cases of recurrence in the group of paroxysmal than persistent AF patients (odds ratio 1.207, 95% confidence interval [CI] 1.048–1.900, *P =* .037). There were no differences in age, sex, body mass index, CHA_2_DS_2_-VASc (congestive heart failure, hypertension, age ≥75 years, diabetes mellitus, prior stroke or transient ischemic attack or thromboembolism, vascular disease, age 65–74 years, sex category) score, left ventricular ejection fraction, LAVI, glomerular filtration rate, comorbidities, or baseline medication regarding AF recurrence (Table 1).

### Biomarker analysis

Biomarker concentrations at baseline and follow-up are shown in Table 2. There were no significant differences in MPO, NT-proANP, ICAM-1, and MMP-2 levels when comparing peripheral venous and coronary sinus samples of the index procedure (all *P* > .05).

**Table 2:** Biomarker concentrations: comparison

	Venous	Coronary sinus	Venous 6-mo follow-up	*P* value (venous vs FU)
MPO, μg/mL	41.9 ± 29.8	37.8 ± 29.4	62.9 ± 37.3	<.001
NT-proANP, pg/mL	469.4 ± 419.1	597.8 ± 476.6	538.9 ± 387.9	.205
ICAM-1, pg/mL	14.4 ± 4.2	12.9 ± 4.6	22.6 ± 13.0	.005
MMP-2, μg/mL	12.3 ± 3.6	9.9 ± 4.3	10.7 ± 20.3	<.001
NT-proBNP, pg/mL	553.9 ± 597.6	—	409.7 ± 443.3	.053
Troponin T (hs), pg/mL	9.5 ± 4.6	—	15.3 ± 19.2	.148
CRP, mg/L	4.2 ± 7.9	—	3.4 ± 3.0	.354

Values are mean ± SD. There were no significant differences when comparing venous with coronary sinus samples (all *P* > .05).

CRP = C-reactive protein; FU = follow-up; hs = high sensitivity; ICAM-1 = intracellular adhesion molecule-1; MMP-2 = matrix metalloproteinase-2; MPO = myeloperoxidase; NT-proANP = N-terminal proatrial natriuretic peptide; NT-proBNP = N-terminal pro–B-type natriuretic peptide.

**Figure 1:**
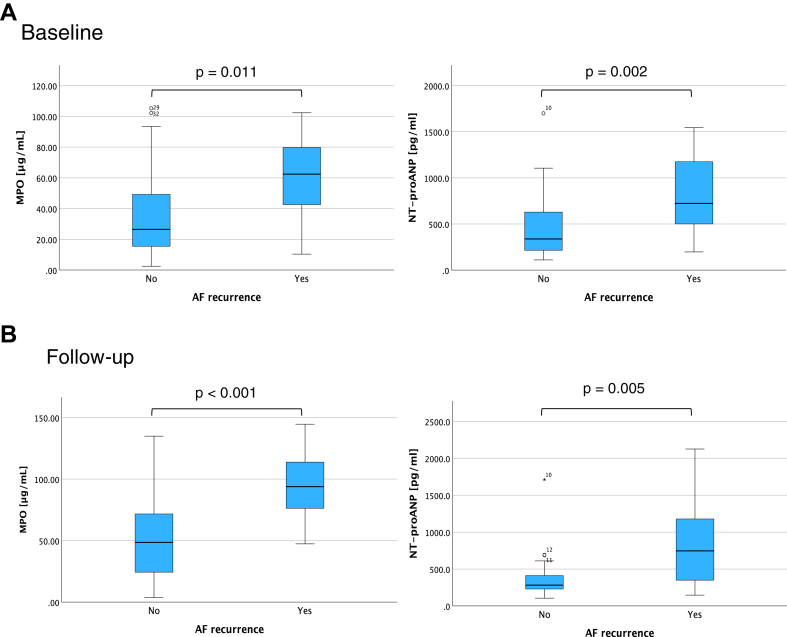
Comparison of myeloperoxidase (MPO) and N-terminal-pro atrial natriuretic peptide (NT-proANP) levels according to atrial fibrillation (AF) recurrence status in patients undergoing redo ablation at baseline (A) and follow-up (B).

Patients with AF recurrence had higher MPO and NT-proANP levels at baseline compared with patients without AF recurrence (Figure 1A, Table 3). Other baseline biomarkers did not differ according to recurrence status.

**Table 3:** Biomarker concentrations according to AF recurrence status

	Overall	No recurrence	Recurrence	*P* value
MPO, μg/mL	33.2 (17.7–61.8)	26.4 (14.7–51.6)	62.4 (40.7–89.1)	.011
NT-proANP, pg/mL	286.2 (231.6–567.1)	281.9 (225.1–429.5)	745.9 (284.7–1,308.0)	<.001
ICAM-1, pg/mL	13.5 (12.4–16.0)	13.5 (12.7–16.1)	12.6 (10.9–16.4)	.349
MMP-2, μg/mL	11.7 (10.4–13.1)	11.6 (10.4–13.4)	12.0 (9.8–14.1)	.254
NT-proBNP, pg/mL	379.5 (144.3–678.5)	377.0 (139.0–604.0)	445.0 (249.0–1,223.0)	.559
Troponin T (hs), pg/ml	9.1 (6.4–11.3)	9.2 (6.9–11.2)	8.3 (5.4–11.9)	.985
CRP, mg/L	2.0 (1.2–3.4)	1.7 (1.1–3.2)	2.6 (1.5–4.7)	.780
FU MPO, μg/mL	56.6 (33.8–89.1)	48.6 (24.3–73.0)	93.9 (71.2–127.9)	.002
FU NT-proANP, pg/mL	401.6 (401.6–783.9)	337.7 (214.4–651.7)	723.00 (499.8–1,262.9)	.005

Values are median (interquartile range). All values denote venous biomarker concentrations (there were no significant differences compared with coronary sinus samples).

AF = atrial fibrillation; CRP = C-reactive protein; FU = follow-up; hs = high sensitivity; ICAM-1 = intracellular adhesion molecule-1; MMP-2 = matrix metalloproteinase-2; MPO = myeloperoxidase; NT-proANP = N-terminal proatrial natriuretic peptide; NT-proBNP = N-terminal pro–B-type natriuretic peptide.

MPO and ICAM-1 increased from baseline to 6 months, whereas MMP-2 decreased over time (Table 2). The increase of MPO was driven by patients with AF recurrence (Figure 1B, Table 3).

### Predictive value

**Figure 2:**
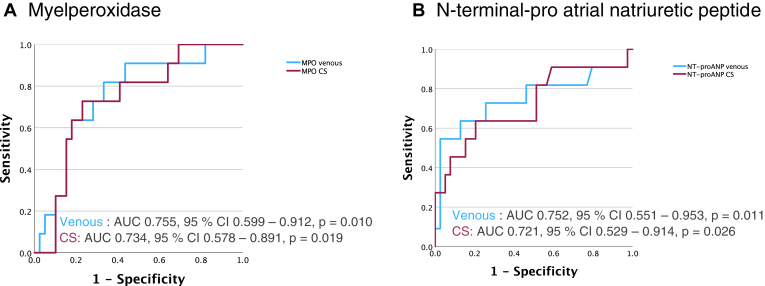
Receiver-operating characteristic curve for (A) myeloperoxidase (MPO) and (B) N-terminal proatrial natriuretic peptide (NT-proANP) levels and atrial fibrillation recurrence status in patients undergoing redo ablation. AUC = area under the curve; CI = confidence interval; CS = coronary sinus.

Receiver-operating characteristic curve analysis showed MPO (venous AUC 0.755, 95% CI 0.599–0.912, *P =* .010; CS 0.734, 95% CI 0.578–0.891, *P =* .019) and NT-proANP (venous AUC 0.752, 95% CI 0.551–0.953, *P =* .011; CS 0.721, 95% CI 0.529–0.914, *P =* .026) as acceptable predictors for intermediate AF recurrence. MMP-2, ICAM-1, and established cardiovascular biomarkers NT-proBNP, troponin T, and C-reactive protein had all AUC <0.600 (Figures 2A and 2B, Table 4).

**Table 4:** Receiver-operating characteristics for established and novel biomarkers for AF recurrence

	Area under the curve	95% confidence interval	*P* value
MPO venous	0.755	0.599–0.912	.010
MPO CS	0.734	0.578–0.891	.019
NT-proANP venous	0.752	0.551–0.953	.011
NT-proANP CS	0.721	0.529–0.914	.026
ICAM-1 venous	0.386	0.146–0.626	.332
ICAM-1 CS	0.487	0.251–0.722	.909
MMP-2 venous	0.570	0.333–0.807	.570
MMP-2 CS	0.503	0.276–0.729	.983
NT-proBNP venous	0.607	0.405–0.809	.281
Troponin T (hs) venous	0.458	0.241–0.675	.673
CRP venous	0.629	0.448–0.810	.092

CRP = C-reactive protein; CS = coronary sinus; hs = high sensitivity; ICAM-1 = intracellular adhesion molecule-1; MMP-2 = matrix metalloproteinase-2; MPO = myeloperoxidase; NT-proANP = N-terminal proatrial natriuretic peptide; NT-proBNP = N-terminal pro–B-type natriuretic peptide.

### Association with AF burden, fibrosis, and LA size

MPO and NT-proANP correlated with AF burden (venous ρ = 0.365, *P =* .005, CS ρ = 0.346, *P =* .007; and venous ρ = 0.362, *P =* .005, CS ρ = 0.309, *P =* .014, respectively).

While MPO was associated with atrial fibrosis in the endocardial map (venous ρ = 0.280, *P =* .024; CS 0.261, *P =* ,033), NT-proANP correlated with LAVI (venous ρ = 0.256, *P =* .037; CS ρ = 0.323, *P =* .011).

Absolute LVAs (<0.5 mV) were higher in patients with recurrence compared with those without (71.9 ± 53.8 cm^2^ vs 40.8 ± 39.5 cm^2^, *P* = .053). There was a significant association between recurrence and fibrosis determined by LVA in relation to LA surface area (ρ = 0.656, *P* < .001) as well as absolute fibrosis (ρ = 0.241, *P =* .049). Receiver-operating characteristic analysis revealed relative LA fibrosis as a good predictor for recurrence of arrythmia (AUC 0.913, 95% CI 0.791–1.000, *P* < .001). Furthermore, MPO was of predictive value for LA fibrosis ≥20% (AUC 0.727, 95% CI 0.540–0.914, *P =* .016), while NT-proANP, ICAM-1, and MMP-2 were not (all AUC <0.600).

## Discussion

AF, by far the most common cardiac arrhythmia, is associated with a high disease burden for patients and represents a challenge for healthcare systems worldwide. Due to demographic changes with an increasingly ageing population, a further increase is expected. Since the implementation of PVI by Haïssaguerre and collagues in 1998,^1^ there has been a steady gain in clinical experience as well as comprehensive development of technical approaches. Nevertheless, there is still a significant number of patients, particularly with persistent and long-standing persistent AF that remain refractory to PVI alone. Therefore, a better understanding of the underlying pathophysiology of AF and patient selection for redo ablations is of great interest.

### Main findings

This is the first study to report that MPO and NT-proANP, both involved in atrial electroanatomic remodeling processes, are of predictive value for AF recurrence in patients undergoing redo ablation. Both biomarkers were associated with AF burden. While MPO correlated with endocardial fibrosis, NT-proANP was associated with LAVI.

### Risk factors for AF

Cardiovascular diseases such as arterial hypertension, heart failure, coronary artery disease, valvular disease, or inflammatory disease are known independent risk factors for AF. In fact, these disorders are believed to be mechanistically involved, as they participate in electrical, contractile, and structural remodeling of atrial tissue.^2^ Literature indicates involvement of intercellular and subcellular mechanisms in atrial tissue alterations as a substrate for AF. Besides electrical abnormalities, mainly based on ion-channel dysfunction and contractile remodeling yielding atrial hypocontractility, structural remodeling owing to atrial dilation and atrial fibrosis has emerged as one of the key aspects in the pathophysiology of AF.^20^ Altogether, those processes can be summarized as atrial electroanatomic remodeling.

### Atrial dilation

Atrial dilation is largely due to pressure and/or volume overload that may result from a variety of causes including heart failure, valvular heart disease, and cardiomyopathies with consequent diastolic dysfunction. All these conditions lead to wall stress with release of atrial natriuretic peptide (ANP). Although ANP is specifically expressed in the atria and is involved in atrial remodeling, B-type natriuretic peptide is associated with mortality and cardiovascular events in AF.^21^ However, ANP may be a mediator and at the same time valuable marker for LA dilation. Further studies suggest that genetic variants of ANP, particularly those leading to enhanced oligomer formation, contribute to the pathogenesis of AF by inducing proarrhythmic metabolic and electrophysiologic effects in atrial myocytes.^22^^,^^23^ In our study, NT-proANP correlated with LA size measured by LAVI and was a predictor for AF recurrence after ablation, which is in line with previous studies.^9^^,^^16^

### Atrial structural remodeling

Atrial structural remodeling is a consequence of increased interstitial fibrosis with cardiac structural alterations and atrial dilation.^24^ Inflammation is one of the leading causes for fibrosis. However, inflammatory processes are complex. In short, cardiac fibroblasts are activated by inflammatory mediators and growth factors associated with systemic inflammatory conditions.^25^ Mediators such as MMPs can be directly activated by leukocytes. Decreasing MMP-2 levels after ablation may be a result of the reduction in inflammation and oxidative stress that follows successful restoration of normal sinus rhythm.^26^ These changes lead to a stabilized atrial environment, decreasing the need for MMP-2 mediated extracellular matrix degradation.

ICAM-1, on the other hand, increased after ablation. The reason for this remains hypothetical: the literature points toward an effect due to ongoing immune responses and structural changes in the atrial tissue.^27^ ICAM-1 expression is involved in remodeling by mediating the interactions between cells and the extracellular matrix.^28^ The remodeling process involves not only the initial inflammatory response, but also the later stages of tissue repair and fibrosis, which can extend over several months well beyond the immediate postprocedural period.^29^

Infiltrated leukocytes account for a large portion of reactive oxygen species and reactive nitrogen species in myocardial tissue via production of superoxide and release of pro-oxidant enzyme systems like MPO. Oxidative stress denotes an unbalanced relation of reactive oxidants and antioxidants.^30^ MPO plays a significant role in AF progression by promoting oxidative stress, inflammation, endothelial dysfunction, and atrial structural remodeling.^31^ Elevated MPO levels have been associated with a switch in AF phenotype and an increased likelihood of AF recurrence after catheter ablation.^32^ Additionally, MPO levels are positively correlated with LA volume in AF patients, suggesting a potential link between MPO, atrial remodeling, and disease progression. According to our data, MPO was an acceptable predictor for AF recurrence. Higher MPO levels, as a potential marker of increased inflammatory activity, were associated with a higher AF burden, and LVAs in the endocardial high-density map. At follow-up MPO levels overall increased slightly, while the effect was mainly attributed to patients with AF recurrence. This may indicate that MPO is a contributor to inflammation, atrial fibrosis, and thus atrial remodeling and underpins its potential role in AF progression and perpetuation.

### Atrial electrical remodeling

Structural atrial alterations promote atrial arrhythmias. These may result from triggers that generate ectopic activity or modifiers of substrate that promote re-entry by affecting action potential duration and refractory periods.^24^ LVAs are regions with reduced electrical activity, often associated with fibrotic changes in the atrial tissue; although the voltage information is also dependent on atrial wall thickness, catheter contact, and atrial rhythm during mapping and do not always accurately reflect atrial fibrosis.^15^ Common thresholds for low voltage are <0.5 mV, as used in our analysis.^17^, ^18^, ^19^ We found a significant association between the amount of LA low voltage and arrhythmia recurrence. The overall percentage of LVAs was high, as well as the average LAVI, indicating a high burden of atrial myopathy in our patient cohort.

LVAs are good predictors for recurrence both in ablation naïve patients and in redo procedures. Their predictive power, though, depends on a detailed map, which is time-consuming and only part of an invasive procedure. Therefore, it would be desirable if there was a surrogate parameter to facilitate LVA assessment in clinical practice.

Our data address 2 important aspects in the complex genesis of AF: NT-proANP is associated with atrial overload and wall stress, and MPO, on the other hand, as a marker for fibrosis, potentially provides further insight into atrial remodeling from a noninvasive perspective. Both biomarkers might be integrated into predictive models alongside other markers of inflammation, cardiac function, and fibrosis to enhance the prediction of AF recurrence. It may be particularly valuable when combined with clinical variables and imaging findings or voltage data from electroanatomic mapping. Assessing MPO and NT-proANP levels could potentially be used for risk stratification in AF patients before repeat ablation, identifying those who may benefit from more aggressive rhythm control strategies.

### Limitations

This is a single-center pilot exploratory study that only included 50 patients, and thus the overall sample size is small. We observed associations but could not establish causal relationships due the study design. The follow-up time was only 6 months.

While NT-proANP is solely expressed in the atria, MPO, ICAM-1, and MMP-2 are known to play a role in cell signaling in different tissues throughout the human body.^21^^,^^33^ Excretion of the latter may therefore reflect not only a cardiac, but also an overall increased state of inflammation and fibrosis and fade diagnostic accuracy. Thus, some studies suggest coronary sinus sampling of cardiac biomarkers potentially superior to peripheral vein puncture because it is responsible for draining most of the blood leaving the myocardium.^34^^,^^35^ However, we did not detect any transcardiac differences from coronary sinus sampling in MPO, NT-proANP, ICAM-1, and MMP-2 levels in this study.

### What this study adds

Our study underlines the role of MPO and NT-proANP in the progression of AF in a cohort of preablated patients with already progressively diseased atria. This might be of help in a better understanding of the pathophysiology of AF, especially with regard to the concept of atrial myopathy, in which MPO is a potential marker of fibrosis and NT-proANP is associated with LA size. Both biomarkers present as acceptable predictors of AF recurrence and could improve patient selection for either further invasive ablation strategies or conservative rhythm control in patients with high risk of recurrence.

## Conclusion

MPO and NT-proANP appear to be acceptable predictors for intermediate recurrence in patients with AF undergoing redo ablation; both were associated with AF burden. While MPO, which is involved in oxidative stress, correlated with endocardial fibrosis, NT-proANP, a marker for atrial wall stress, was associated with LAVI. Future studies should investigate their potential and pathophysiological role in AF perpetuation and progression in a larger cohort.
